# Prevalences of hyperuricemia and electrolyte abnormalities in patients with chronic kidney disease in Japan: A nationwide, cross-sectional cohort study using data from the Japan Chronic Kidney Disease Database (J-CKD-DB)

**DOI:** 10.1371/journal.pone.0240402

**Published:** 2020-10-15

**Authors:** Tadashi Sofue, Naoki Nakagawa, Eiichiro Kanda, Hajime Nagasu, Kunihiro Matsushita, Masaomi Nangaku, Shoichi Maruyama, Takashi Wada, Yoshio Terada, Kunihiro Yamagata, Ichiei Narita, Motoko Yanagita, Hitoshi Sugiyama, Takashi Shigematsu, Takafumi Ito, Kouichi Tamura, Yoshitaka Isaka, Hirokazu Okada, Kazuhiko Tsuruya, Hitoshi Yokoyama, Naoki Nakashima, Hiromi Kataoka, Kazuhiko Ohe, Mihoko Okada, Naoki Kashihara

**Affiliations:** 1 Division of Nephrology and Dialysis, Department of Cardiorenal and Cerebrovascular Medicine, Kagawa University, Kagawa, Japan; 2 Division of Cardiology, Nephrology, Respiratory and Neurology, Department of Internal Medicine, Asahikawa Medical University, Asahikawa, Japan; 3 Medical Science, Kawasaki Medical School, Kurashiki, Japan; 4 Department of Nephrology and Hypertension, Kawasaki Medical School, Kurashiki, Japan; 5 Department of Epidemiology, Johns Hopkins Bloomberg School of Public Health, Baltimore, Maryland, United States of America; 6 Division of Nephrology and Endocrinology, University of Tokyo Graduate School of Medicine, Tokyo, Japan; 7 Department of Nephrology, Nagoya University Graduate School of Medicine, Nagoya, Japan; 8 Department of Nephrology and Laboratory Medicine, Kanazawa University, Kanazawa, Japan; 9 Department of Endocrinology, Metabolism and Nephrology, Kochi Medical School, Kochi University, Kochi, Japan; 10 Department of Nephrology, Faculty of Medicine, University of Tsukuba, Tsukuba, Japan; 11 Division of Clinical Nephrology and Rheumatology, Niigata University Graduate School of Medical and Dental Sciences, Niigata, Japan; 12 Department of Nephrology, Graduate School of Medicine, Kyoto University, Kyoto, Japan; 13 Department of Human Resource Development of Dialysis Therapy for Kidney Disease, Okayama University Graduate School of Medicine, Dentistry and Pharmaceutical Sciences, Okayama, Japan; 14 Department of Nephrology, Wakayama Medical University, Wakayama, Japan; 15 Division of Nephrology, Faculty of Medicine, Shimane University, Izumo, Japan; 16 Department of Medical Science and Cardiorenal Medicine, Yokohama City University Graduate School of Medicine, Yokohama, Japan; 17 Department of Nephrology, Osaka University Graduate School of Medicine, Suita, Japan; 18 Department of Nephrology, Faculty of Medicine, Saitama Medical University, Saitama, Japan; 19 Department of Integrated Therapy for Chronic Kidney Disease, Kyushu University, Fukuoka, Japan; 20 Department of Nephrology, Nara Medical University, Kashihara, Japan; 21 Department of Nephrology, Kanazawa Medical University School of Medicine, Ishikawa, Japan; 22 Medical Information Center, Kyushu University Hospital, Fukuoka, Japan; 23 Faculty of Health Science and Technology, Kawasaki University of Medical Welfare, Kurashiki, Japan; 24 Department of Healthcare Information Management, The University of Tokyo Hospital, Tokyo, Japan; 25 Institute of Health Data Infrastructure for All, Tokyo, Japan; International University of Health and Welfare, School of Medicine, JAPAN

## Abstract

**Background:**

The Japan Chronic Kidney Disease Database (J-CKD-DB) is a nationwide clinical database of patients with chronic kidney disease (CKD) based on electronic health records. The objective of this study was to assess the prevalences of hyperuricemia and electrolyte abnormalities in Japanese patients with CKD.

**Methods:**

In total, 35,508 adult outpatients with estimated glomerular filtration rates of 5–60 ml/min/1.73 m^2^ in seven university hospitals were included this analysis. The proportions of patients with CKD stages G3b, G4, and G5 were 23.5%, 7.6%, and 3.1%, respectively.

**Results:**

Logistic regression analysis showed that prevalence of hyperuricemia was associated with CKD stages G3b (adjusted odds ratio [95% confidence interval]: 2.12 [1.90–2.37]), G4 (4.57 [3.92–5.32]), and G5 (2.25 [1.80–2.80]). The respective prevalences of hyponatremia, hypercalcemia, hyperphosphatemia, and narrower difference between serum sodium and chloride concentrations were elevated in patients with CKD stages G3b, G4, and G5, compared with those prevalences in patients with CKD stage G3a. The prevalences of hyperkalemia were 8.3% and 11.6% in patients with CKD stages G4 and G5, respectively. In patients with CKD stage G5, the proportions of patients with optimal ranges of serum uric acid, potassium, corrected calcium, and phosphate were 49.6%, 73.5%, 81.9%, and 56.1%, respectively.

**Conclusions:**

We determined the prevalences of hyperuricemia and electrolyte abnormalities in Japanese patients with CKD using data from a nationwide cohort study.

## Introduction

A reduction in estimated glomerular filtration rate (eGFR) <60 ml/min/1.73 m^2^ is regarded as a sign of chronic kidney disease (CKD), as are structural or functional renal abnormalities [1]. Approximately 13% of Japanese adults are estimated to have CKD [2]. Cross-sectional estimates of the prevalence of CKD in the United States range from 1.5% to 15.6% [3]. CKD is categorized on the basis of eGFR and the degree of proteinuria [4], and is reportedly a major risk factor for cardiovascular disease [5]. Moreover, the socioeconomic impacts of CKD are important global problems [6].

Important complications of CKD include hyperuricemia and electrolyte abnormalities (e.g., hyperkalemia, hypocalcemia, hyperphosphatemia, and metabolic acidosis), as well as renal anemia [7, 8]. Although it remains controversial, asymptomatic hyperuricemia has been reported as a risk factor for eGFR decline in patients with CKD [9, 10]. Hyperkalemia and hyperphosphatemia are known risk factors for cardiovascular disease and mortality in patients with CKD [11–16].

The prevalences of hyperuricemia and electrolyte abnormalities in patients with CKD have been reported in clinical and epidemiological studies [8–11, 13–18]. Recently, analyses of real-world conditions of patients with CKD were performed based on electronic health records [12, 19–22]; these studies included analyses of electrolyte abnormality prevalences among patients with CKD [12]. However, electronic health record analyses of the prevalences of hyperuricemia and electrolyte abnormalities according to CKD G category have been limited.

The Japan Chronic Kidney Disease Database (J-CKD-DB) is a large-scale, nationwide comprehensive clinical database of patients with CKD based on electronic health records data from participating university hospitals [23, 24]. Using a standardized method for information exchange (Standardized Structured Medical Information eXchange; SS-MIX2) [25], the J-CKD-DB efficiently compiles clinical data of patients with CKD across hospitals, regardless of differences in electronic health records systems. The J-CKD-DB cohort includes automatically extracted data regarding all patients with CKD in Japan. Therefore, the J-CKD-DB enables investigations of real-world CKD practices in Japan through both cross-sectional and prospective studies.

The aim of this study was to assess the real-world prevalences of hyperuricemia and electrolyte abnormalities in Japanese patients with CKD by using automatically extracted data from a nationwide, large-scale cohort study in Japan.

## Materials and methods

### Study setting and participants

The J-CKD-DB is a multicenter, automatically extracted comprehensive database of patients with CKD from 21 university hospitals in Japan (UMIN trial number, UMIN000026272) [23, 24]. The inclusion criteria for the J-CKD-DB are as follows: 1) age ≥18 years; 2) proteinuria ≥1+ (dipstick test) and/or eGFR <60 ml/min/1.73 m^2^, where eGFR is calculated using the Japanese eGFR equation: eGFR (ml/min/1.73 m^2^) = 194 × serum creatinine value ^-1.094^ × age—^0.287^ (× 0.739 [for women]) [26]. All data elements are extracted in a semiautomatic manner via the SS-MIX2 format and stored in the Multipurpose Clinical Data Registry System [23, 27]. Patients undergoing renal replacement therapy (i.e., hemodialysis, peritoneal dialysis, and kidney transplantation) are manually omitted.

In this study, we performed an observational cross-sectional investigation. To exclude most patients with acute kidney injury, we omitted inpatients from this analysis [24]. We identified 39,121 adult outpatients with at least one measurement of serum creatinine in seven university hospitals from January 1, 2014 to December 31, 2014. We included 35,508 patients with CKD based on outpatient laboratory data that indicated eGFR of 5–60 ml/min/1.73 m^2^ [24, 28, 29].

### Data collection

CKD G categories were defined as follows: stage G3a, eGFR of ≥45 and <60 ml/min/1.73 m^2^; stage G3b, eGFR of ≥30 and <45 ml/min/1.73 m^2^; stage 4, eGFR of ≥15 and <30 ml/min/1.73 m^2^; and stage 5, eGFR <15 ml/min/1.73 m^2^ [1, 24]. eGFR5 categories were divided into 11 subgroups based on eGFR ranges of 5 ml/min/1.73 m^2^ each, as performed in a previous study [24].

Data for this analysis that were obtained from the SSMIX-2 system included age, sex, eGFR, dipstick proteinuria, hemoglobin, serum albumin, total cholesterol, sodium, potassium, chloride, calcium, phosphate, and C-reactive protein. The lowest value of eGFR during the study period was selected as the eGFR value for analysis. Data without related eGFR values were extracted for 1 week before or 1 week after an eGFR value was recorded [23]. The difference between serum sodium and chloride concentrations was determined as serum Na–serum Cl (mEq/L) [30]. Serum corrected calcium level was calculated using Payne's correction formula: measured serum calcium level + (4 –serum albumin level) (mg/dl) [31]. Mean serum uric acid (UA) level was analyzed according to G category, eGFR5 category, age, and sex strata. Mean electrolyte levels were analyzed according to G category and eGFR5 category.

The optimal range for serum UA level was defined as <6.0 mg/dl, based on Japanese clinical practice guidelines during the study period [32]. The optimal ranges of electrolytes were defined as serum sodium >135 mEq/L to <145 mEq/L [33], serum potassium >3.5 mEq/L to <5.5 mEq/L [34], serum corrected calcium >8.4 mg/dl to <10.3 mg/dl [35], and serum phosphate >2.5 mg/dl to <4.5 mg/dl [35]. Hyperuricemia was defined as serum UA level ≥7.0 mg/dl [32]. Electrolyte disorders were defined as follows: hypernatremia and hyponatremia as serum sodium levels of ≥145 mEq/L and ≤135 mEq/L, respectively; hyperkalemia and hypokalemia as serum potassium levels of ≥5.5 mEq/L and ≤3.5 mEq/L, respectively; hypercalcemia and hypocalcemia as serum corrected calcium levels of ≥10.3 mg/dl and ≤8.4 mg/dl, respectively; and hyperphosphatemia and hypophosphatemia as serum phosphate levels of ≥4.5 mg/dl and ≤2.5 mg/dl, respectively [33–35]. Narrower serum Na–Cl level was defined as ≤30 mEq/L [30]. To identify the effect of season on the prevalence of hyperuricemia, participants were divided into four subgroups according to the date of blood sample collection: winter (December, January, and February), spring (March, April, and May), summer (June, July, and August), and autumn (September, October, and November).

### Ethics statement

The J-CKD-DB study was comprehensively approved by the ethics committee of Kawasaki Medical School and Japanese Society of Nephrology (JSN-28) and individually approved by local committees of participating university hospitals (Kagawa University Hospital, Asahikawa Medical University Hospital, University of Tokyo Graduate School of Medicine, Nagoya University Hospital, Kanazawa University Hospital, Kochi University Hospital, University of Tsukuba Hospital, Niigata University Hospital, Kyoto University Hospital, Okayama University Hospital, Wakayama Medical University Hospital, Shimane University Hospital, Yokohama City University Hospital, Osaka University Hospital, Saitama Medical University Hospital, Nara Medical University Hospital, Kanazawa Medical University Hospital and Kyushu University Hospital). The study was conducted in accordance with the ethical principles of the World Medical Association Declaration of Helsinki. Because all data had been fully anonymized prior to retrieval for this study, informed consent was obtained in the form of opt-out on the website of each participating university hospital. Patients who declined to participate in the J-CKD-DB were not registered.

### Statistical analysis

Values are presented as medians with interquartile intervals, means with standard deviations, or counts with percentages, as appropriate. Distributions of variables were evaluated by histogram, quantile-quantile plot, and the Kolmogorov–Smirnov test. Clinical variables were compared between groups using the χ^2^ test for categorical variables and Student’s t-test, one-way analysis of variance, two-way analysis of variance, or the Mann–Whitney U test for continuous variables. Tukey's multiple comparison test was used for post hoc corrections for multiple comparisons. All data were statistically analyzed using IBM SPSS Advanced Statistics, version 27.0 (IBM Corp., Armonk, NY, USA), and p<0.05 was considered to indicate significant differences.

To identify independent associations of G category with the prevalences of hyperuricemia and electrolyte abnormalities, multivariable logistic regression models were used. The adjusted odds ratios and corresponding two-sided 95% confidence intervals of the predictors were determined. Model 1 (including age, sex, G grade, A grade, hemoglobin level, albumin level, C-reactive protein, hypernatremia, hypokalemia, and season of sample collection as covariates) was used for hyperuricemia; model 2 (including age, sex, G grade, A grade, hemoglobin level, albumin level, and C-reactive protein as covariates) was used for hypernatremia, hyponatremia, hyperkalemia, hypokalemia, narrower serum Na–Cl level, hyperphosphatemia, and hypophosphatemia; model 3 (including age, sex, G grade, A grade, hemoglobin level, and C-reactive protein as covariates) was used for hypercalcemia and hypocalcemia; and model 4 (including age, sex, G grade, A grade, albumin level, hemoglobin level, and narrower serum Na–Cl level as covariates) was used for hyperkalemia.

## Results

### Baseline characteristics of enrolled outpatients

The sampling rate stratification of the 35,508 patients, according to G category strata, is shown in Table 1. Serum UA and potassium were collected in 73.0% and 80.5% of patients with CKD, respectively; serum calcium and phosphate were collected in 41.9% and 30.4% of patients with CKD, respectively. However, the sampling rate of each electrolyte, especially serum calcium and phosphate, increased in accordance with the progression of G category.

**Table 1:** Sampling rate stratification according to G category strata.

		UA	Na	K	Cl	Ca	P
G3a	%	70.1	61.7	76.7	54.6	38.1	25.7
G3b	%	75.5*	68.8*	85.4*	60.5*	43.2*	31.9*
G4	%	84.6*	74.5*	93.1*	68.6*	58.1*	50.6*
G5	%	85.4*	75.0*	94.7*	71.3*	71.8*	67.2*
Total	%	73.0^†^	64.8	80.5^†^	57.6^†^	41.9^†^	30.4^†^

Sampling rate is expressed as % of each population.

Abbreviations: UA, serum uric acid; Na, serum sodium; K, serum potassium; Cl, serum chloride; Ca, serum calcium; P, serum phosphate.

*:p<0.05 vs. G3a

^†^:p<0.05 vs. Na.

Baseline characteristics of enrolled outpatients are shown in Table 2. The median age was 72 [interquartile interval, 64–79] years, 54.5% were men, and median eGFR was 50.0 [interquartile interval, 40.9–55.6] ml/min/1.73 m^2^. The numbers of patients with CKD stages G3a, G3b, G4, and G5 were 23,333 (65.7%), 8,357 (23.5%), 2,710 (7.6%), and 1,108 (3.1%), respectively. The mean hemoglobin level (standard deviation) of all patients was 13.0 (1.9) g/dl. Stratification of patients according to eGFR category, age, and sex strata is shown in S1 Table. Serum UA and electrolyte status stratifications according to G category strata are shown in Table 3.

**Table 2:** General characteristics of study cohort upon enrollment.

*N*	35,508
Age (years)	72.0 [64.0–79.0]
Age category	
18–45 years	1,173 (3.3%)
45–64 years	7,966 (22.4%)
65–74 years	11,628 (32.7%)
75–84 years	11,309 (31.8%)
≥ 85 years	3,432 (9.7%)
Gender: men	19,360 (54.5%)
eGFR (ml/min/1.73m^2^)	50.0 [40.9–55.6]
eGFR stage	
G3a	23,333 (65.7%)
G3b	8,357 (23.5%)
G4	2,710 (7.6%)
G5	1,108 (3.1%)
Dipstick proteinuria	Overall: 15,442
(-)	9,357 (60.6%)
(±)	2,295 (14.9%)
(1+)	1,849 (12.0%)
(2+)	1,277 (8.3%)
(3+)	598 (3.9%)
(4+)	66 (0.4%)
Hemoglobin (g/dL)	13.02 (1.88)
Serum albumin (g/dL)	4.04 (0.47)
Serum uric acid (mg/dL)	6.05 (1.49)
Serum total cholesterol (mg/dL)	187.9 (38.3)
Serum sodium (mEq/L)	141.0 (2.84)
Serum potassium (mEq/L)	4.41 (0.54)
Serum chloride (mEq/L)	104.6 (3.34)
Serum calcium (mg/dL)	9.08 (0.54)
Serum phosphate (mg/dL)	3.52 (0.94)
Serum C-reactive protein (mg/dL)	0.10 [0.04–0.28]

Continuous variables are expressed as median [interquartile interval] or mean (standard deviation). Factors are expressed as n (%).

Abbreviation: eGFR, estimated glomerular filtration rate.

**Table 3:** Serum uric acid and electrolyte status stratification according to G category strata.

		UA	Na	K	Cl	Na-Cl	cCa	P
	unit	(mg/dl)	(mEq/L)	(mEq/L)	(mEq/L)	(mEq/L)	(mg/dl)	(mg/dl)
G3a	mean	5.80	141.3	4.33	104.6	36.8	9.23	3.40
	SD	1.33	2.51	0.44	2.82	2.1	0.42	0.69
G3b	mean	6.36*	140.8*	4.46*	104.6	36.3*	9.29	3.44
	SD	1.47	2.99	0.59	3.49	2.6	0.50	0.95
G4	mean	6.94*	140.4*	4.68*	105.3*	35.2*	9.30	3.70*
	SD	1.78	3.62	0.73	4.65	3.4	0.63	1.22
G5	mean	6.20*	139.8*	4.71*	103.9*	36.0*	9.19	4.44*
	SD	2.23	4.17	0.76	5.57	4.3	0.77	1.39

Values are expressed as mean and SD.

*:p<0.05 vs. G3a.

Abbreviations: UA, serum uric acid; Na, serum sodium; K, serum potassium; Cl, serum chloride; Na-Cl, difference between serum sodium and chloride concentrations; cCa, serum corrected calcium; P, serum phosphate; SD, standard deviation.

### Association between serum UA level and eGFR

**Fig 1:**
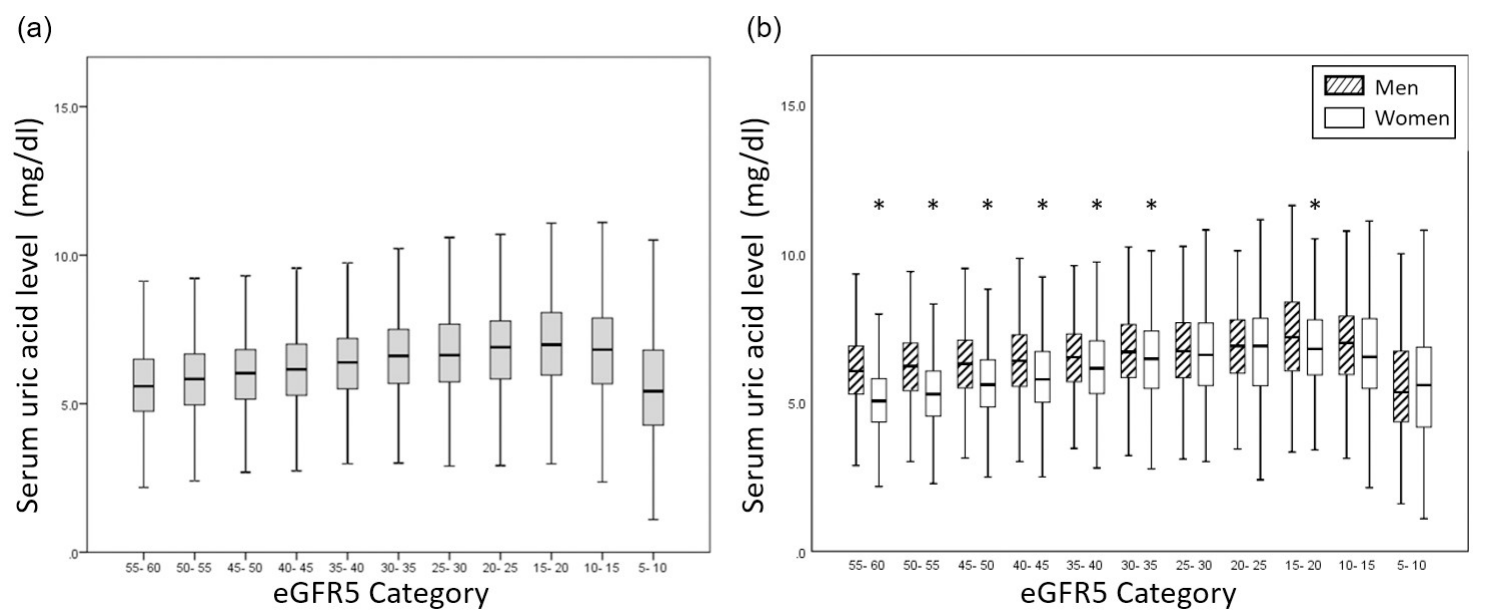
Mean serum uric acid level according to eGFR5 category. (a) All participants; (b) Men and women shown separately. Abbreviation: eGFR, estimated glomerular filtration rate. *, p<0.05.

**Fig 2:**
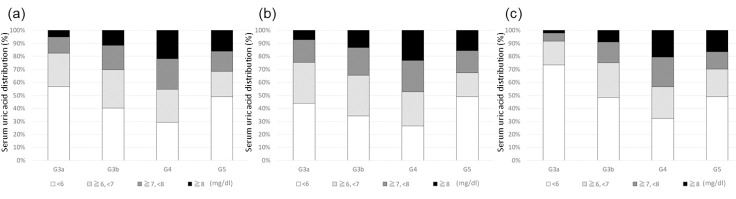
Serum uric acid distribution according to G category. (a) All participants; (b) Men; and (c) Women.

Mean serum UA levels according to eGFR5 category and sex strata are shown in Fig 1. The mean serum UA level increased in accordance with the progression of eGFR5 category in both male and female patients. However, the mean serum UA level was lower in patients with an eGFR category of ≥5 and <10 ml/min/1.73 m^2^ than in patients with an eGFR category of ≥10 and <15 ml/min/1.73 m^2^. The mean serum UA level was higher in male patients than in female patients among patients with an eGFR category of >30 ml/min/1.73 m^2^, and an eGFR of ≥15 and <20 ml/min/1.73 m^2^. Serum UA distribution according to G category and sex strata is shown in Fig 2. The proportion of patients with high serum UA level increased in accordance with the progression of G category in both male and female patients, except among patients with CKD stage G5.

The mean serum UA levels according to G category, age, and sex strata are shown in S2 Table. The mean serum UA level gradually increased in accordance with the progression of G category, except among patients with CKD stage G5. Differences in UA level according to sex and age disappeared with the progression of G category.

### Prevalences of hyperuricemia and rates of patients in the optimal range for serum UA level

The prevalences of hyperuricemia according to G category, age, and sex strata are shown in S3 Table. The prevalence of hyperuricemia increased in accordance with the progression of G category. However, the prevalence of hyperuricemia was lower in patients with CKD stage G5 than in patients with CKD stage G4. Differences in prevalence of hyperuricemia according to sex and age disappeared with the progression of G category.

Logistic regression analysis using model 1 showed that the adjusted odds ratios and 95% confidence intervals for the prevalences of hyperuricemia in patients with CKD stages G3b, G4, and G5 were 2.12 [1.90–2.37], 4.57 [3.92–5.32], and 2.25 [1.80–2.80], respectively (Table 4). Hypernatremia and hypokalemia were also independent risk factors for greater prevalence of hyperuricemia. There was no significant seasonal effect on the prevalence of hyperuricemia.

**Table 4:** Adjusted odds ratios and 95% confidence intervals for hyperuricemia.

covariate		hyperuricemia (UA ≥ 7.0 mg/dl)
G grade	G3a	1
	G3b	2.12 [1.90–2.37]
	G4	4.57 [3.92–5.32]
	G5	2.25 [1.80–2.80]
hypernatremia (Na ≥ 145 mEq/L)		1.42 [1.15–1.76]
hypokalemia (K ≤ 3.5 mEq/L)		1.43 [1.10–1.86]
season of sample collection.	Winter	1
	Spring	0.93 [0.82–1.07]
	Summer	1.12 [0.99–1.27]
	Autumn	1.04 [0.91–1.19]

Covariates: age, sex, G grade, A grade, hemoglobin level, albumin level, C-reactive protein, hypernatremia (Na ≥145 mEq/L), hypokalemia (K ≤3.5 mEq/L), and season of sample collection.

Adjusted odds ratios and 95% confidence intervals were analyzed by logistic regression analysis using each of the above factors as covariates.

Abbreviations: UA, serum uric acid; Na, serum sodium; K, serum potassium.

The rates of serum UA level within the optimal range according to G category, age, and sex strata are shown in Table 5; these were 29.6% in patients with CKD stage G4 and 49.6% in patients with CKD stage G5. The rates of serum UA level within the optimal range in patients with CKD stage G4 were 27.0% in male patients and 32.6% in female patients.

**Table 5:** Rates of UA level within optimal range (UA ≤6.0 mg/dl) according to G category, age, and sex strata.

		18–44 Y	45–64 Y	65–74 Y	75–84 Y	85+ Y	men	women	Subtotal
G3a	%	46.2^†^	52.4	55.7^†^	62.0^†^	68.2^†^	44.4	73.8^‡^	57.2
G3b	%	34.2*	34.2*	39.3^†^*	43.0^†^*	48.4^†^*	34.7*	48.9^‡^*	40.8*
G4	%	25.8*	27.1*	28.0*	33.2^†^*	29.4*	27.0*	32.6^‡^*	29.6*
G5	%	49.4	52.9	51.5	45.5*	40.4*	49.7*	49.4*	49.6*

Numbers of patients within optimal ranges are expressed as % of each population.

*:p<0.05 vs. G3a

†:p<0.05 vs. 45–64 Y

^‡^:p<0.05 vs. men.

Abbreviation: UA, serum uric acid.

### Associations between electrolyte disorders and eGFR

**Fig 3:**
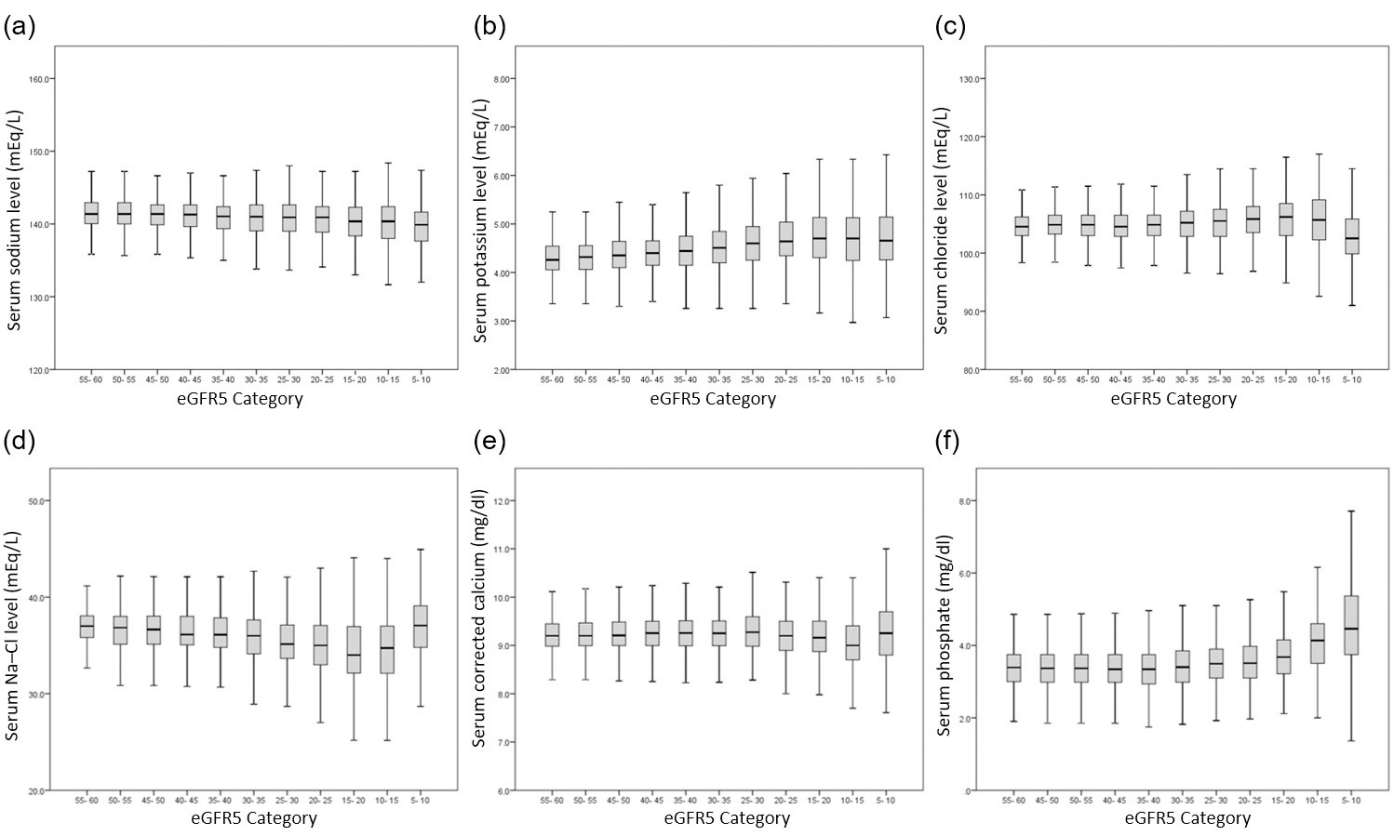
Mean serum electrolyte levels according to eGFR5 category. (a) Serum sodium level; (b) Serum potassium level; (c) Serum chloride level; (d) Serum Na–Cl level; (e) Serum corrected calcium level; and (f) Serum phosphate level. Abbreviations: Serum Na-Cl level, difference between serum sodium and chloride concentrations; eGFR, estimated glomerular filtration rate.

The mean serum levels of each electrolyte according to eGFR5 category are shown in Fig 3. The mean serum potassium and phosphate levels increased in accordance with the progression of eGFR5 category, whereas the mean serum chloride and corrected calcium levels decreased in accordance with the progression of eGFR5 category. However, mean serum chloride and corrected calcium levels were higher in patients with an eGFR category of ≥5 and <10 ml/min/1.73 m^2^ than in patients with an eGFR category of ≥10 and <15 ml/min/1.73 m^2^.

**Fig 4:**
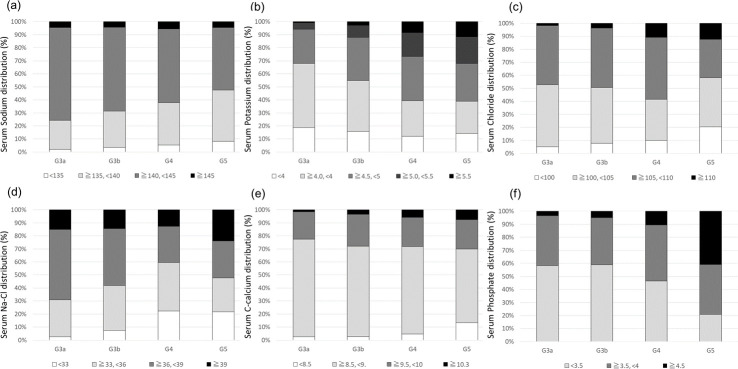
Serum electrolyte distribution according to G category. (a) Serum sodium distribution (%); (b) Serum potassium distribution (%); (c) Serum chloride distribution (%); (d) Serum Na–Cl distribution (%); (e) Serum corrected calcium distribution (%); and (f) Serum phosphate distribution (%). Abbreviations: Serum Na-Cl level, difference between serum sodium and chloride concentrations; C-calcium, corrected calcium.

The distributions of electrolytes according to G category are shown in Fig 4. The rates of patients with high serum potassium and phosphate levels increased in accordance with the progression of G category; the rate of patients with low serum sodium level also increased in accordance with the progression of G category. Furthermore, the rates of patients with low and high serum corrected calcium levels both increased in accordance with the progression of G category.

### Prevalence of electrolyte disorders and ratio of optimal range of electrolytes

The prevalences of electrolyte disorders according to G category are shown in S4 Table. The prevalences of hyponatremia, hyperkalemia, hypercalcemia, hyperphosphatemia, and narrower serum Na–Cl level gradually increased in accordance with the progression of G category. Notably, the prevalence of hypokalemia was elevated in patients with CKD stage G5, while the prevalence of hypocalcemia was elevated in patients with CKD stages G4 and G5. In contrast, the prevalence of hypophosphatemia was reduced in patients with CKD stages G4 and G5.

The adjusted odds ratios and 95% confidence intervals for electrolyte disorders according to G category strata are shown in Table 6. Logistic regression analysis demonstrated that the respective prevalences of hyponatremia, hyperkalemia, hypercalcemia, hyperphosphatemia, and narrower serum Na–Cl level were elevated in patients with CKD stages G3b, G4, and G5, compared with those prevalences in patients with CKD stage G3a. The prevalence of hypocalcemia was elevated only in patients with CKD stage G5. Further logistic regression analysis (model 4) showed that a narrower serum Na–Cl level was associated with the prevalence of hyperkalemia (adjusted odds ratio, 3.18 [95% confidence interval, 2.13–4.74]), independent of G category (S5 Table).

**Table 6:** Adjusted odds ratios and 95% confidence intervals for electrolyte disorders according to G category strata.

		Na ≤ 135 mEq/L*	Na ≥ 145 mEq/L*	K ≤ 3.5 mEq/L*
G3a	Reference	1	1	1
G3b	AOR [95% CI]	1.37 [1.08–1.73]	0.98 [0.79–1.12]	0.94 [0.74–1.19]
G4	AOR [95% CI]	1.67 [1.25–2.25]	1.05 [0.78–1.42]	0.57 [0.39–0.84]
G5	AOR [95% CI]	1.77 [1.23–2.55]	0.98 [0.62–1.54]	0.98 [0.65–1.48]
		K ≥ 5.5 mEq/L*	cCa ≤ 8.4 mg/dl^†^	cCa ≥ 10.3 mg/dl^†^
G3a	Reference	1	1	1
G3b	AOR [95% CI]	3.09 [2.32–4.12]	1.04 [0.72–1.51]	2.56 [1.87–3.51]
G4	AOR [95% CI]	9.38 [6.94–12.7]	0.95 [0.59–1.51]	3.89 [2.69–5.64]
G5	AOR [95% CI]	9.33 [6.55–13.3]	3.12 [2.09–4.66]	4.82 [3.14–7.40]
		P ≤ 2.5 mg/dl*	P ≥ 4.5 mg/dl*	Na-Cl ≤ 30 mEq/L*
G3a	Reference	1	1	1
G3b	AOR [95% CI]	1.12 [0.89–1.41]	1.36 [1.02–1.81]	4.79 [2.46–9.33]
G4	AOR [95% CI]	0.44 [0.30–0.65]	2.69 [2.01–3.61]	13.5 [6.96–26.1]
G5	AOR [95% CI]	0.30 [0.18–0.52]	15.6 [11.8–20.7]	15.2 [7.52–30.6]

Covariates:

*age, sex, G grade, A grade, hemoglobin level, albumin level, and C-reactive protein

^†^age, sex, G grade, A grade, hemoglobin level, and C-reactive protein.

Adjusted odds ratios and 95% confidence intervals were analyzed by logistic regression analysis with each of the above factors as covariates.

Abbreviations: AOR, adjusted odds ratio; CI, confidence interval; UA, serum uric acid; Na, serum sodium; K, serum potassium; Cl, serum chloride; Na-Cl, difference between serum sodium and chloride concentrations; cCa, serum corrected calcium; P, serum phosphate.

The rates of patients within optimal ranges for serum potassium, corrected calcium, and phosphate levels according to G category are shown in Table 7. The rates of patients within optimal ranges for serum corrected calcium and phosphate levels decreased in accordance with the progression of G category. In patients with CKD stage G5, the rates of patients within optimal ranges for serum potassium, corrected calcium, and phosphate levels were 73.5%, 81.9%, and 56.1%, respectively.

**Table 7:** Rates of patients within optimal ranges for serum potassium, corrected calcium, and phosphate levels according to G category.

		K ≥ 4.0, ≤ 5.5 mEq/L	cCa ≥ 8.4, ≤ 10.3 mg/dl	P ≥ 2.5, ≤ 4.5 mg/dl
G3a	%	79.7	97.2	90.9
G3b	%	81.0*	94.6*	87.9*
G4	%	79.1	91.0*	86.2*
G5	%	73.5*	81.9*	56.1*

Numbers of patients within optimal ranges are expressed as % of each population.

Abbreviations: K, serum potassium; cCa, serum corrected calcium; P, serum phosphate.

*:p<0.05 vs. G3a.

## Discussion

In this study, we demonstrated the prevalences of hyperuricemia and electrolyte abnormalities in 35,508 Japanese patients with CKD by using automatically extracted data from a nationwide, large-scale cohort study in Japan. The J-CKD-DB study enables large-scale analysis directly linked to the electronic medical record by using the SS-MIX2 standard, and is an important precursor to large-scale analyses of complications in Japanese patients with CKD.

Previous studies reported the prevalences of electrolyte abnormalities in patients with CKD who were treated by nephrologists [14, 17, 30]. Here, we examined the sampling rate of electrolytes in an electronic health record study cohort, which was not limited to patients receiving treatment from nephrologists. The sampling rate of each electrolyte was low among patients with CKD stage G3 and increased with the progression of G category. A previous electronic health record study showed that 85% of patients with CKD underwent measurement of serum potassium level [36], which was similar to our findings. Notably, the results of this study were analyzed among patients for whom electrolyte data were collected.

In our cohort study of Japanese patients with CKD, we found that the prevalences of hyperuricemia were higher among men and younger patients with CKD stage G3a; however, sex and age differences disappeared with the progression of G category. The prevalences of hyperuricemia were higher in patients with CKD stages G3b, G4, and G5, compared with patients with CKD stage G3a; however, the prevalence of hyperuricemia was lower in patients with CKD stage G5 than in patients with CKD stage G4, regardless of age and sex. Similar results were obtained regarding the rate of patients within the optimal range for serum uric acid level. It was difficult to determine whether these results were due to enhanced use of uric acid-lowering drugs or to the inaccurate inclusion of patients receiving dialysis, because the current analysis did not allow for the evaluation of drugs. Future analyses of prescriptions (HOT reference code [37]) are needed to assess the extent to which drug-based interventions are performed among Japanese patients with CKD. However, the present study showed that hypernatremia and hypokalemia were associated with the prevalence of hyperuricemia, independent of G category; this finding may indicate an effect of diuretics. Participants from whom blood samples were collected in the summer tended to have a higher prevalence of hyperuricemia, but this was not statistically significant. Additional studies are needed to determine how fluid volume and salt reduction affect uric acid levels, in addition to the influences of oral urate-lowering medication and diuretics.

To the best of our knowledge, this is the first large-scale evaluation of electrolyte abnormalities in patients with CKD, using automatically extracted data in Japan. With the progression of G category, the prevalences of hyperkalemia, hypocalcemia, and hyperphosphatemia increased, as did the prevalences of hyponatremia, hypokalemia, narrower serum Na–Cl level, and hypercalcemia. The prevalence of hyponatremia increased with the progression of G category, compared with CKD stage G3b, implying that the use of diuretics may contribute to hyponatremia. An increase in the prevalence of hypercalcemia was observed with the progression of G category. The prevalence of hypocalcemia is generally presumed to increase with the progression of CKD. However, a recent report showed an increased prevalence of hypercalcemia in patients with CKD [38]. Importantly, the use of vitamin D preparations may contribute to hypercalcemia. Furthermore, differences in the prevalences of hypernatremia and hypokalemia, due to the progression of G category, disappeared after adjustment for confounding factors. The prevalences of electrolyte abnormalities in relation to G category progression are likely to change over time with the advent of new drugs. Therefore, a longitudinal study of patients with CKD is necessary.

The rates of patients within optimal ranges for potassium, calcium, and phosphate decreased with the progression of G category. This result is presumably because the sampling rate of each electrolyte increased with the progression of G category. The percentage of patients within the optimal range for each electrolyte was assessed in accordance with the clinical practice guidelines for the study period. The achievement rates and quality indicators of clinical practice guidelines should be assessed in further longitudinal studies.

Although hyperkalemia is a well-recognized complication of CKD, a previous study reported that the prevalences of hyperkalemia (14%–20%) and hypokalemia (12%–18%) were similar [39]. In patients with CKD, hypokalemia and hyperkalemia have both been reported to enhance the risk of mortality [16]. CKD severity, use of medications such as renin-angiotensin-aldosterone system inhibitors and diuretics, and dietary potassium intake are major determinants of serum potassium concentration in patients with CKD. The results of our electronic health record study showed that the prevalence of hypokalemia was not elevated among Japanese patients with CKD.

Metabolic acidosis is known to be associated with worsening kidney function in patients with CKD [40]. Bicarbonate supplementation has been reported to slow the progression of CKD among patients with CKD stage G4 who exhibit a low serum bicarbonate concentration [41]. The serum Na–Cl level is not equivalent to the serum bicarbonate level, but has been described as a simple parameter for assessment of acid–base status [30, 42]. A previous study reported that a narrower serum Na–Cl level was associated with worsening kidney function in patients with CKD stage G4 [30]. In this study, a narrower serum Na–Cl level was a risk factor for hyperkalemia, independent of G category. Although the rate of sodium bicarbonate administration was not determined, metabolic acidosis is a possible cause of hyperkalemia, in addition to renal dysfunction.

Several limitations of our study should be noted, in relation to its cross-sectional design. First, we were unable to obtain information concerning the prevalence of gout, administration or types and dosages of drugs, and differences in practice patterns between nephrologists and other clinicians because we could not readily convert some of the local codes regarding medical care and tests to standardized codes, despite the availability of reference mapping tables. More accurate analysis might be achieved by information regarding the use and frequency of medications such as renin-angiotensin-aldosterone system inhibitors, beta blockers, urate-lowering drugs, diuretics, potassium adsorbents, vitamin D drugs, and anti-hyperphosphatemia drugs. Parathyroid hormone and magnesium levels were also collected, but could not be analyzed because local code settings were not yet available. Second, we were unable to obtain information regarding the cause of CKD, body mass index, presence or absence of diabetes and cardiovascular diseases, or blood pressure levels because these elements are not included in the SS-MIX2 system; thus, it was difficult to investigate research questions related to these variables.

In conclusion, we demonstrated the prevalences of hyperuricemia and electrolyte abnormalities in Japanese patients with CKD by using automatically extracted data from a nationwide, large-scale cohort study in Japan. We also assessed the influences of G category and other factors on the prevalences of hyperuricemia and electrolyte abnormalities. Further prospective investigations including drugs and health insurance claim data, as well as analyses regarding quality indicators for clinical practice guidelines, may contribute to improvements in the quality of care for patients with CKD.

## Supporting information

S1 Table Participant stratification according to eGFR category, age, and sex strata. (PDF)

S2 Table Serum uric acid status according to G category, age, and sex strata. (PDF)

S3 Table Prevalence of hyperuricemia according to G category, age, and sex strata. (PDF)

S4 Table Prevalences of electrolyte abnormalities according to G category strata. (PDF)

S5 Table Adjusted odds ratios and 95% confidence intervals for hyperkalemia (K ≥5.5 mEq/L). (PDF)

## References

[1] National Kidney Foundation. K/DOQI clinical practice guidelines for chronic kidney disease: evaluation, classification, and stratification. Am J Kidney Dis. 2002;39: S1–266. Available: http://www.ncbi.nlm.nih.gov/pubmed/11904577 11904577

[2] Imai E, Horio M, Watanabe T, Iseki K, Yamagata K, Hara S, et al Prevalence of chronic kidney disease in the Japanese general population. Clin Exp Nephrol. 2009;13: 621–630. 10.1007/s10157-009-0199-x 19513802

[3] Zhang Q-L, Rothenbacher D. Prevalence of chronic kidney disease in population-based studies: Systematic review. BMC Public Health. 2008;8: 117 10.1186/1471-2458-8-117 18405348 PMC2377260

[4] Levey AS, Eckardt K-U, Tsukamoto Y, Levin A, Coresh J, Rossert J, et al Definition and classification of chronic kidney disease: a position statement from Kidney Disease: Improving Global Outcomes (KDIGO). Kidney Int. 2005;67: 2089–100. 10.1111/j.1523-1755.2005.00365.x 15882252

[5] Sarnak MJ, Levey AS, Schoolwerth AC, Coresh J, Culleton B, Hamm LL, et al Kidney disease as a risk factor for development of cardiovascular disease: a statement from the American Heart Association Councils on Kidney in Cardiovascular Disease, High Blood Pressure Research, Clinical Cardiology, and Epidemiology and Prevention. Circulation. 2003;108: 2154–69. 10.1161/01.CIR.0000095676.90936.80 14581387

[6] Travers K, Martin A, Khankhel Z, Boye KS, Lee LJ. Burden and management of chronic kidney disease in Japan: Systematic review of the literature. Int J Nephrol Renovasc Dis. 2012 10.2147/IJNRD.S30894 23319870 PMC3540912

[7] Ohno I. Relationship between hyperuricemia and chronic kidney disease. Nucleosides, Nucleotides and Nucleic Acids. 2011;30: 1039–1044. 10.1080/15257770.2011.611484 22132954

[8] Barbour SJ, Er L, Djurdjev O, Karim MA, Levin A. The prevalence of hematologic and metabolic abnormalities during chronic kidney disease stages in different ethnic groups. Kidney Int. 2008;74: 108–114. 10.1038/ki.2008.151 18432185

[9] Momoki K, Kataoka H, Moriyama T, Mochizuki T, Nitta K. Hyperuricemia as a predictive marker for progression of nephrosclerosis: Clinical assessment of prognostic factors in biopsy-proven arterial/arteriolar nephrosclerosis. J Atheroscler Thromb. 2017;24: 630–642. 10.5551/jat.37523 27784849 PMC5453688

[10] Petreski T, Ekart R, Hojs R, Bevc S. Asymptomatic hyperuricemia and cardiovascular mortality in patients with chronic kidney disease who progress to hemodialysis. Int Urol Nephrol. 2019;51: 1013–1018. 10.1007/s11255-019-02154-w 31020628

[11] Einhorn LM, Zhan M, Hsu VD, Walker LD, Moen MF, Seliger SL, et al The frequency of hyperkalemia and its significance in chronic kidney disease. Arch Intern Med. 2009;169: 1156–1162. 10.1001/archinternmed.2009.132 19546417 PMC3544306

[12] Kashihara N, Kohsaka S, Kanda E, Okami S, Yajima T. Hyperkalemia in Real-World Patients Under Continuous Medical Care in Japan. Kidney Int Reports. 2019;4: 1248–1260. 10.1016/j.ekir.2019.05.018 31517144 PMC6734103

[13] Kestenbaum B, Sampson JN, Rudser KD, Patterson DJ, Seliger SL, Young B, et al Serum phosphate levels and mortality risk among people with chronic kidney disease. J Am Soc Nephrol. 2005;16: 520–528. 10.1681/ASN.2004070602 15615819

[14] Voormolen N, Noordzij M, Grootendorst DC, Beetz I, Sijpkens YW, Van Manen JG, et al High plasma phosphate as a risk factor for decline in renal function and mortality in pre-dialysis patients. Nephrol Dial Transplant. 2007;22:2909–2916. 10.1093/ndt/gfm286 17517792

[15] Inker LA, Grams ME, Levey AS, Coresh J, Cirillo M, Collins JF, et al Relationship of Estimated GFR and Albuminuria to Concurrent Laboratory Abnormalities: An Individual Participant Data Meta-analysis in a Global Consortium. Am J Kidney Dis. 2019;73: 206–217. 10.1053/j.ajkd.2018.08.013 30348535 PMC6348050

[16] Korgaonkar S, Tilea A, Gillespie BW, Kiser M, Eisele G, Finkelstein F, et al Serum potassium and outcomes in CKD: Insights from the RRI-CKD cohort study. Clin J Am Soc Nephrol. 2010;5: 762–769. 10.2215/CJN.05850809 20203167 PMC2863985

[17] Mandai S, Kanda E, Iimori S, Naito S, Noda Y, Kikuchi H, et al Association of serum chloride level with mortality and cardiovascular events in chronic kidney disease: the CKD-ROUTE study. Clin Exp Nephrol. 2017;21: 104–111. 10.1007/s10157-016-1261-0 27039905

[18] Bennett WM, Porter GA. Efficacy and safety of metolazone in renal failure and the nephrotic syndrome. J Clin Pharmacol. 1973;13: 357–364. 10.1002/j.1552-4604.1973.tb00224.x 4579972

[19] Kim LG, Cleary F, Wheeler DC, Caplin B, Nitsch D, Hull SA, et al How do primary care doctors in England and Wales code and manage people with chronic kidney disease? Results from the National Chronic Kidney Disease Audit. Nephrol Dial Transplant. 2018;33: 1373–1379. 10.1093/ndt/gfx280 29045728 PMC6070084

[20] Iwagami M, Tomlinson LA, Mansfield KE, Casula A, Caskey FJ, Aitken G, et al Validity of estimated prevalence of decreased kidney function and renal replacement therapy from primary care electronic health records compared with national survey and registry data in the United Kingdom. Nephrol Dial Transplant. 2017;32: ii142–ii150. 10.1093/ndt/gfw318 28201668 PMC5410977

[21] Su G, Xu H, Marrone G, Lindholm B, Wen Z, Liu X, et al Chronic kidney disease is associated with poorer in-hospital outcomes in patients hospitalized with infections: Electronic record analysis from China. Sci Rep. 2017;7: 11530 10.1038/s41598-017-11861-2 28912532 PMC5599500

[22] Jolly SE, Navaneethan SD, Schold JD, Arrigain S, Sharp JW, Jain AK, et al Chronic kidney disease in an electronic health record problem list: quality of care, ESRD, and mortality. Am J Nephrol. 2014;39: 288–96. 10.1159/000360306 24714513 PMC4056768

[23] Nakagawa N, Sofue T, Kanda E, Nagasu H, Matsushita K, Nangaku M, et al J-CKD-DB: a nationwide multicentre electronic health record-based chronic kidney disease database in Japan. Sci Rep. 2020;10: 7351 10.1038/s41598-020-64123-z 32355258 PMC7192920

[24] Sofue T, Nakagawa N, Kanda E, Nagasu H, Matsushita K, Nangaku M, et al Prevalence of anemia in patients with chronic kidney disease in Japan: A nationwide, cross-sectional cohort study using data from the Japan Chronic Kidney Disease Database (J-CKD-DB). PLoS ONE. 2020;15(7):e0236132 10.1371/journal.pone.0236132 32687544 PMC7371174

[25] Kimura M, Nakayasu K, Ohshima Y, Fujita N, Nakashima N, Jozaki H, et al SS-MIX: a ministry project to promote standardized healthcare information exchange. Methods Inf Med. 2011;50: 131–9. 10.3414/ME10-01-0015 21206962

[26] Matsuo S, Imai E, Horio M, Yasuda Y, Tomita K, Nitta K, et al Revised equations for estimated GFR from serum creatinine in Japan. Am J Kidney Dis. 2009;53: 982–92. 10.1053/j.ajkd.2008.12.034 19339088

[27] Sugiyama T, Miyo K, Tsujimoto T, Kominami R, Ohtsu H, Ohsugi M, et al Design of and rationale for the Japan Diabetes compREhensive database project based on an Advanced electronic Medical record System (J-DREAMS). Diabetol Int. 2017;8: 375–382. 10.1007/s13340-017-0326-y 30603343 PMC6224921

[28] Chapter 1: Definition and classification of CKD. Kidney Int Suppl. 2013;3: 19–62. 10.1038/kisup.2012.64 25018975 PMC4089693

[29] Ohta M, Babazono T, Uchigata Y, Iwamoto Y. Comparison of the prevalence of chronic kidney disease in Japanese patients with Type 1 and Type 2 diabetes. Diabet Med. 2010;27: 1017–23. 10.1111/j.1464-5491.2010.03049.x 20722675

[30] Maruta Y, Hasegawa T, Yamakoshi E, Nishiwaki H, Koiwa F, Imai E, et al Association between serum Na-Cl level and renal function decline in chronic kidney disease: results from the chronic kidney disease Japan cohort (CKD-JAC) study. Clin Exp Nephrol. 2019;23: 215–222. 10.1007/s10157-018-1631-x 30168046 PMC6510908

[31] Payne RB, Little AJ, Williams RB, Milner JR. Interpretation of Serum Calcium in Patients with Abnormal Serum Proteins. Br Med J. 1973;4: 643–646. 10.1136/bmj.4.5893.643 4758544 PMC1587636

[32] Yamanaka H. Japanese guideline for the management of hyperuricemia and gout: Second edition. Nucleosides, Nucleotides and Nucleic Acids. 2011;30: 1018–1029. 10.1080/15257770.2011.596496 22132951

[33] Kamel Sand Halperin M, Fluid, Electrolyte and Acid-Base Physiology: A Problem-Based Approach, 5th edition, Elsevier

[34] Evidence-based Clinical Practice Guideline for CKD 2013. Clin Exp Nephrol. 2014; 18, 346–423. 10.1007/s10157-014-0949-2 24817136

[35] Fukagawa M, Yokoyama K, Koiwa F, Taniguchi M, Shoji T, Kazama JJ, et al Clinical practice guideline for the management of chronic kidney disease-mineral and bone disorder. Ther Apher Dial. 2013;17: 247–288. 10.1111/1744-9987.12058 23735142

[36] Nakhoul GN, Huang H, Arrigain S, Jolly SE, Schold JD, Nally J V., et al Serum Potassium, End-Stage Renal Disease and Mortality in Chronic Kidney Disease. Am J Nephrol. 2015;41: 456–463. 10.1159/000437151 26228532 PMC4686260

[37] Kawazoe Y, Imai T, Ohe K. A Querying Method over RDF-ized Health Level Seven v2.5 Messages Using Life Science Knowledge Resources. JMIR Med Informatics. 2016;4: e12 10.2196/medinform.5275 27050304 PMC4837294

[38] Seng JJB, Tan YLC, Lim RW, Ng HTS, Lee PH, Wong J. Prevalence and risk factors for hypercalcemia among non-dialysis patients with chronic kidney disease-mineral and bone disorder. Int Urol Nephrol. 2018;50: 1871–1877. 10.1007/s11255-018-1906-x 29882003

[39] Gilligan S, Raphael KL. Hyperkalemia and Hypokalemia in CKD: Prevalence, Risk Factors, and Clinical Outcomes. Advances in Chronic Kidney Disease. W.B. Saunders; 2017 pp. 315–318. 10.1053/j.ackd.2017.06.004 29031358

[40] Dobre M, Yang W, Chen J, Drawz P, Hamm LL, Horwitz E, et al Association of serum bicarbonate with risk of renal and cardiovascular outcomes in CKD: A report from the Chronic Renal Insufficiency Cohort (CRIC) study. Am J Kidney Dis. 2013;62: 670–678. 10.1053/j.ajkd.2013.01.017 23489677 PMC3701754

[41] De Brito-Ashurst I, Varagunam M, Raftery MJ, Yaqoob MM. Bicarbonate supplementation slows progression of CKD and improves nutritional status. J Am Soc Nephrol. 2009;20: 2075–2084. 10.1681/ASN.2008111205 19608703 PMC2736774

[42] Havlin J, Matousovic K, Schück O. Sodium-Chloride Difference as a Simple Parameter for Acid-Base Status Assessment. American Journal of Kidney Diseases. W.B. Saunders; 2017 pp. 707–708. 10.1053/j.ajkd.2016.12.019 28285873

