# Supplementary material for: Prevalences of hyperuricemia and electrolyte abnormalities in patients with chronic kidney disease in Japan: A nationwide, cross-sectional cohort study using data from the Japan Chronic Kidney Disease Database (J-CKD-DB)
Source: PLoS One. 2020 Oct 15;15(10):e0240402. doi: 10.1371/journal.pone.0240402 (PMC7561156; doi:10.1371/journal.pone.0240402)
Supplement: S1 Table — (PDF) [file pone.0240402.s001.pdf]

S1 Table: Participant Stratification According to eGFR Category, Age, and Sex Strata.

	eGFR category			
	G3a	G3b	G4	G5
eGFR range, mL/min/1.73 m^2	45–59	30–44	15–29	<15
n	23,333	8,357	2,710	1,108
Prevalence	65.7%	23.5%	7.6%	3.1%
Age strata				
18–44 years	718<br>(61.2%)	223<br>(19.0%)	131<br>(11.2%)	101<br>(8.6%)
45–64 years	5,815<br>(73.0%)	1,330<br>(16.7%)	489<br>(6.1%)	332<br>(4.2%)
65–74 years	8,146<br>(70.1%)	2,472<br>(21.3%)	688<br>(5.9%)	322<br>(2.8%)
75–84 years	6,994<br>(61.8%)	3,131<br>(27.7%)	908<br>(8.0%)	276<br>(2.4%)
85+ years	1,660<br>(48.4%)	1,201<br>(35.0%)	494<br>(14.4%)	77<br>(2.2%)
Sex strata				
Men	12,661<br>(65.4%)	4,614<br>(23.8%)	1,434<br>(7.4%)	651<br>(3.4%)
Women	10,672<br>(66.1%)	3,743<br>(23.2%)	1,276<br>(7.9%)	457<br>(2.8%)

Abbreviation: eGFR, estimated glomerular filtration rate;
