# Supplementary material for: Prevalences of hyperuricemia and electrolyte abnormalities in patients with chronic kidney disease in Japan: A nationwide, cross-sectional cohort study using data from the Japan Chronic Kidney Disease Database (J-CKD-DB)
Source: PLoS One. 2020 Oct 15;15(10):e0240402. doi: 10.1371/journal.pone.0240402 (PMC7561156; doi:10.1371/journal.pone.0240402)
Supplement: S2 Table — (PDF) [file pone.0240402.s002.pdf]

S2 Table: Serum Uric Acid Status According to G Category, Age, and Sex Strata.

Total		18-44 Y	45-64 Y	65-74 Y	75-84 Y	85+ Y	men	women	subtotal
G3a	mean	6.21†	5.96	5.83†	5.66†	5.48†	6.18	5.31‡	5.80
	SD	1.63	1.39	1.29	1.25	1.32	1.28	1.22	1.33
G3b	mean	6.59*	6.59*	6.44*†	6.26*†	6.14*†	6.55*	6.11*‡	6.36*
	SD	1.72	1.57	1.42	1.41	1.46	1.44	1.46	1.47
G4	mean	7.03*	7.05*	6.93*	6.80*†	7.08*	7.05*	6.82*‡	6.94*
	SD	1.86	1.73	1.69	1.75	1.96	1.80	1.76	1.78
G5	mean	6.43	6.03	6.13*	6.28*	6.68*†	6.22	6.16*	6.20*
	SD	2.99	2.01	2.27	2.12	2.03	2.29	2.14	2.23

serum uric acid level (mg/dl) were expressed as mean and standard deviation (SD) and analyzed by one-way ANOVA.

*:$p<0.05$ vs. G3a,†: $p<0.05$ vs. 45-64Y, ‡:$p<0.05$ vs. men.
