# Supplementary material for: Prevalences of hyperuricemia and electrolyte abnormalities in patients with chronic kidney disease in Japan: A nationwide, cross-sectional cohort study using data from the Japan Chronic Kidney Disease Database (J-CKD-DB)
Source: PLoS One. 2020 Oct 15;15(10):e0240402. doi: 10.1371/journal.pone.0240402 (PMC7561156; doi:10.1371/journal.pone.0240402)
Supplement: S3 Table — (PDF) [file pone.0240402.s003.pdf]

S3 Table: Prevalence of Hyperuricemia According to G Category, Age, and Sex Strata.

		18-44 Y	45-64 Y	65-74 Y	75-84 Y	85+ Y	men	women	subtotal
G3a	%	27.1†	21.7	17.8†	13.6†	12.8†	24.7	8.3‡	17.6
G3b	%	33.7	35.3*	31.4†*	28.6†*	25.6†*	34.5*	24.8‡*	30.3*
G4	%	49.2*	49.7*	46.3*	41.3†*	45.6*	47.2*	43.4*	45.4*
G5	%	31.5	30.2*	29.4*	33.0*	42.1*	32.7*	29.9*	31.5*

The prevalence rate of hyperuricemia was expressed as % of each population and analyzed by chi-square test.

*:p<0.05 vs. G3a,†: p<0.05 vs. 45-64Y, ‡:p<0.05 vs. men

Abbreviation: UA, serum uric acid level
