# Supplementary material for: Prevalences of hyperuricemia and electrolyte abnormalities in patients with chronic kidney disease in Japan: A nationwide, cross-sectional cohort study using data from the Japan Chronic Kidney Disease Database (J-CKD-DB)
Source: PLoS One. 2020 Oct 15;15(10):e0240402. doi: 10.1371/journal.pone.0240402 (PMC7561156; doi:10.1371/journal.pone.0240402)
Supplement: S4 Table — (PDF) [file pone.0240402.s004.pdf]

S4 Table: Prevalences of Electrolyte Abnormalities According to G Category Strata.

		Na ≤ 135mEq/L	Na ≥ 145 mEq/L	K ≤ 3.5 mEq/L	K ≥ 5.5 mEq/L	Na-Cl ≤ 30 mEq/L
G3a	%	2.1%	4.5%	2.1%	0.7%	0.2%
G3b	%	3.7%*	4.3%	2.3%	2.7%*	1.0%*
G4	%	5.6%*	5.5%*	2.2%	8.3%*	4.6%*
G5	%	9.1%*	4.5%	4.2%*	11.6%*	7.5%*
		cCa ≤ 8.4 mg/dl	cCa ≥ 10.3 mg/dl	P ≤ 2.5 mg/dl	P ≥ 4.5 mg/dl	
G3a	%	1.4%	1.4%	5.7%	3.4%	
G3b	%	1.9%	3.5%*	7.2%*	4.8%*	
G4	%	3.1%*	5.8%*	3.4%*	10.4%*	
G5	%	10.7%*	7.4%*	3.1%*	40.8%*	

The prevalence rate was expressed as % of each population.

Abbreviation: Na, serum sodium; K, serum potassium, Cl, serum chloride; Na-Cl, the difference between serum sodium and chloride concentratios; cCa, serum corrected calcium, P, serum phosphate

*:p<0.05 vs. G3a.
