# Supplementary material for: Prevalences of hyperuricemia and electrolyte abnormalities in patients with chronic kidney disease in Japan: A nationwide, cross-sectional cohort study using data from the Japan Chronic Kidney Disease Database (J-CKD-DB)
Source: PLoS One. 2020 Oct 15;15(10):e0240402. doi: 10.1371/journal.pone.0240402 (PMC7561156; doi:10.1371/journal.pone.0240402)
Supplement: S5 Table — (PDF) [file pone.0240402.s005.pdf]

S5 Table: Adjusted Odds Ratios and 95% Confidence Intervals for Hyperkalemia (K ≥5.5 mEq/L).

		hyperkalemia (K ≥5.5 mEq/L)
Narrower serum Na-Cl		3.18 [2.13-4.74]
G grade	G3a	1
	G3b	3.57 [2.66-4.80]
	G4	10.7 [7.81-14.6]
	G5	11.8 [8.23-17.0]

Covariate: age, sex, G grade, A grade, Alb, Hb, Narrower serum Na-Cl

The adjusted odds ratio and 95% confidence intervals were analyzed by logistic regression analysis with each of the above factors as covariates.

Abbreviation: Na, serum sodium; K, serum potassium, Cl, serum chloride; Na-Cl, the difference between serum sodium and chloride concentratios;
